# Altered Coupling between Motion-Related Activation and Resting-State Brain Activity in the Ipsilesional Sensorimotor Cortex after Cerebral Stroke

**DOI:** 10.3389/fneur.2017.00339

**Published:** 2017-07-19

**Authors:** Jianping Hu, Juan Du, Qiang Xu, Fang Yang, Fanyong Zeng, Xi-jian Dai, Xiaoxue Liu, Guangming Lu, Zhiqiang Zhang

**Affiliations:** ^1^Department of Medical Imaging, Jinling Hospital, Nanjing Clinical School, Southern Medical University, Nanjing, China; ^2^Department of Radiology, The First Affiliated Hospital, Fujian Medical University, Fuzhou, China; ^3^Department of Medical Imaging, Jinling Hospital, School of Medicine, Nanjing University, Nanjing, China; ^4^Department of Neurology, Jinling Hospital, School of Medicine, Nanjing University, Nanjing, China; ^5^State Key Laboratory of Analytical Chemistry for Life Science, Nanjing University, Nanjing, China

**Keywords:** stroke, motor recovery, functional magnetic resonance imaging, resting-state functional connectivity, motion-related activation

## Abstract

Functional connectivity maps using resting-state functional magnetic resonance imaging (rs-fMRI) can closely resemble task fMRI activation patterns, suggesting that resting-state brain activity may predict task-evoked activation or behavioral performance. However, this conclusion was mostly drawn upon a healthy population. It remains unclear whether the predictive ability of resting-state brain activity for task-evoked activation would change under different pathological conditions. This study investigated dynamic changes of coupling between patterns of resting-state functional connectivity (RSFC) and motion-related activation in different stages of cerebral stroke. Twenty stroke patients with hand motor function impairment were involved. rs-fMRI and hand motion-related fMRI data were acquired in the acute, subacute, and early chronic stages of cerebral stroke on a 3-T magnetic resonance (MR) scanner. Sixteen healthy participants were enrolled as controls. For each subject, an activation map of the affected hand was first created using general linear model analysis on task fMRI data, and then an RSFC map was determined by seeding at the peak region of hand motion activation during the intact hand task. We then measured the extent of coupling between the RSFC maps and motion-related activation maps. Dynamic changes of the coupling between the two fMRI maps were estimated using one-way repeated measures analysis of variance across the three stages. Moreover, imaging parameters were correlated with motor performances. Data analysis showed that there were different coupling patterns between motion-related activation and RSFC maps associating with the affected motor regions during the acute, subacute, and early chronic stages of stroke. Coupling strengths increased as the recovery from stroke progressed. Coupling strengths were correlated with hand motion performance in the acute stage, while coupling recovery was negatively correlated with the recovery outcome of hand motion performance in the early chronic stages. Couplings between RSFC and motion-related activation were dynamically changed with stroke progression, which suggested changes in the prediction of resting-state brain activity for task-evoked brain activity in different pathological states. The changes in coupling strength between these two types of brain activity implicate a reparative mechanism of brain injury and may represent a biomarker for predicting motor recovery in cerebral stroke.

## Introduction

Blood oxygenation level-dependent (BOLD) functional magnetic resonance imaging (fMRI), as a non-invasive imaging technique, has become an effective tool for investigating the human brain, both in a clinical context and a research context. Conventional task-based fMRI can depict the topological pattern of stimulus or task-evoked brain activation through specific experimental designs, and has been used to estimate the function, and localize the eloquent regions of brain diseases (1, 2). Over recent years, resting-state fMRI (rs-fMRI), by measuring the features of low-frequency BOLD fluctuations, has grown to become an alternative approach to explore human brain function (3–5). Studies have demonstrated that functional connectivity maps with associated seed regions, as provided by rs-fMRI, show a consistent pattern with corresponding task fMRI activation maps for many functional brain systems (6, 7). Thus, rs-fMRI can be used for clinical estimations, such as presurgical location of functional areas in patients with brain tumors (8–12); this approach has several advantages, such as ease of use, and the fact that this technique is free of accuracy limitations resulting from difficulties associated with patient task performance (13).

The underlying physiological mechanism of resting-state brain activity is one of the main research priorities in the neuroimaging field. Although there is a large body of studies that have interrogating the relationship between resting-state brain activity and associated task-evoked brain activity using fMRI (7, 14, 15), low-frequency spontaneous brain activity has been interpreted as unconstrained behavior or conscious mentation (7). Moreover, the pattern of resting-state spontaneous brain activity is also regarded as a potential predictor for brain activation (16–18), and an individual’s task performance or behavior (19). However, these interpretations were mostly based on studies involving a healthy population. It is known that BOLD activity represents physiological effects arising from blood flow, oxygenation, and energetic metabolism (20). It is conceivable that pathologies of brain will affect BOLD effects and, consequently, will affect the relationship between resting-state brain activity and the associated task-evoked brain activity. However, no previous studies have used fMRI to investigate the changing patterns of coupling between these two types of activity under different pathological conditions.

In this study, we carried out a longitudinal fMRI study on patients with cerebral stroke at different stages of progression. By observing the relationship between resting-state brain activity and task-evoked activation associated with hand motion, we were able to estimate the changes in dynamic coupling following cerebral stroke. We hypothesized that the coupling pattern between rs-fMRI and task-based fMRI would be influenced by the specific pathological condition involved, and that this would involve a time-dependent change following the recovery of motor function. Confirmation of these hypotheses would provide new insight into our understanding of the relationship between rs-fMRI and task-based fMRI under pathological conditions.

## Materials and Methods

### Clinical and Image Data Acquisition

This study was approved by the local Ethical Committee and all subjects provided informed written consent. A total of 20 acute ischemic stroke patients with hand motor impairment (17 men, 3 women; age: 50.95 ± 11.40 years, age range: 30–71 years; 6 with right-side deficit) were enrolled in the Inpatient Department of Jingling Hospital (Nanjing, China) between January 2015 and July 2016. The inclusion criteria were as follows: (1) first-ever ischemic stroke, (2) unilateral hand motor deficit, (3) symptom onset <7 days, (4) age between 18 and 80 years, and (5) single stroke lesion located in the territory of the middle cerebral artery. The exclusion criteria were as follows: (1) hemorrhagic stroke, (2) bilateral stroke lesions on MRI, (3) language or cognitive deficits sufficient to affect informed consent, (4) other orthopedic, neurological, or psychiatric disease substantially affecting the arm, (5) contraindications for MRI examination, and (6) recurrent stroke during follow-up. In addition, 16 healthy subjects (12 men, 4 women; age: 54.25 ± 2.51 years; age range: 35–68 years) were included as an age-matched control group.

Clinical and imaging data were obtained at three time points: acute stage (3.75 ± 1.62 days after the onset of stroke; day range: 1–7), subacute stage (9.95 ± 2.16 days after stroke; day range: 7–14 days), early chronic stage (98.3 ± 8.39 days after stroke; day range: 87–116 days). The hand motor function of each patient was assessed using the Upper Limb Fugl-Meyer Assessment (UL-FMA) score, which ranges from 0 (complete hemiplegia) to 66 (normal performance) for the upper extremities. Mean UL-FMA scores at each time point were 36.1 ± 15.65 (range: 6–59), 43.6 ± 17.18 (range: 7–62), and 55.60 ± 11.49 (range: 27–66), respectively. The mean size of stroke lesions was 3.36 ± 2.08 ml (range: 0.61–6.44 ml). Demographic and clinical characteristics of stroke patients are shown in Table 1. Specific pieces of information for each patient are shown in Table S1 in Supplementary Material. A lesion map across all patients during the acute stage is shown in Figure S1 in Supplementary Material.

**Table 1:** Demographic and clinical data of stoke patients.

Patients (*n* = 20)	Acute stage	Subacute stage	Early chronic stage
Age (years)	50.95 ± 11.40 (32–71)	–	–
Sex (male)	17/20	–	–
Lesion side (left)	14/20	–	–
Lesion volume (ml)	3.36 ± 2.08 (0.61–6.44)	–	–
Days after stroke	3.75 ± 1.62 (1–7)	9.95 ± 2.16 (7–14)	98.3 ± 8.39 (87–116)
UL-FMA	36.1 ± 15.65 (6–59)	43.6 ± 17.18 (7–62)	55.60 ± 11.49 (27–66)

*UL-FMA, Upper Limb Fugl-Meyer Assessment*.

All MR images were acquired using a 3.0-T whole-body scanner (Discovery MR 750, GE Healthcare, Milwaukee, WI, USA) with a 32-channel phased-array head coil. Participants were placed on the scanner gantry in a head-first supine position using plastic holders to minimize head motion and ear plugs to reduce scanner noise. A high-resolution 3D T1-weighted structural image was obtained in the transverse orientation using a 3D-BRAVO sequence with the following parameters: TR = 8.2 ms, TE = 3.2 ms, flip angle = 12°, FOV = 220 mm × 220 mm, matrix = 256 × 256, slice thickness = 1.0 mm. Resting-state and a subsequent task-fMRI data were acquired using a gradient-echo EPI sequence with the following scan parameters: TR = 2,000 ms, TE = 30 ms, flip angle = 80°, FOV = 240 mm × 240 mm, matrix = 64 × 64, slice thickness = 3.0 mm, no gap, slice number = 43. The scan range covered the whole brain tissue extending from the frontal–parietal cortex to the lower parts of the cerebellum. Each resting-state scan consisted of 205 volumes and lasted 6 min 50 s. Participants were instructed to keep their eyes closed, stay as motionless as possible, think of nothing in particular, and not fall asleep. For task fMRI scans, participants underwent a block-designed hand motion task. There were three types of blocks: a task block addressing performance in the affected hand (the right hand for healthy subjects), a task block addressing performance in the unaffected hand (the left hand for healthy subjects), and a control block with rest. During a task block, the participant was instructed to tap their fingers using their affected or unaffected hand with a frequency of 1 Hz. Each task was repeated five times pseudo-randomly and a total of 15 blocks were included in one session. Each block lasted 20 s and one task scan lasted 5 min, consisting of 150 volumes.

### MRI Data Processing

MRI data preprocessing was conducted using SPM8^1^ and Data Processing Assistant for Resting-State fMRI (21). In order to enlarge sample size and permit the analysis of all patients as one homogeneous group, the imaging data from patients with right hemispheric lesions were flipped from right to left along the midsagittal plane. Thus, after flipping, the left hemisphere corresponded to the ipsilesional side and the right hemisphere corresponded to the contralesional side in all patients.

The first five images were removed to ensure steady-state longitudinal magnetization for rs-fMRI data. Then, slice-timing and realignment were performed for task- and rs-fMRI data. Translation or rotation parameters in any given data set did not exceed ±1.5 mm or ±1.5°. To avoid tissue misclassification caused by the infarcted tissue during image normalization, a cost-function method was used to remove the influence of lesions (22, 23). A lesion mask was created on individual 3D T1-weighted structural images by two radiologists (Hu JP and Zeng FY), using DWI images as a guide. Then, the individual images were normalized into the standard brain template of the Montreal Neurological Institute by using a 12-parameter affine transformation with non-linear adjustments and were resampled to 3 mm × 3 mm × 3 mm voxel size. Spatial smooth (FWHM = 8 mm) was then performed upon the normalized functional images.

For task-data, and for each subject, the task conditions were convolved with the canonical hemodynamic response function and modeled as regressors in the general linear model. For each subject, the BOLD activation of each hand motion was estimated using a first-level analysis of variance (ANOVA) with task condition as the main factor: affected hand finger tapping task (AHFT) vs. control and unaffected hand finger tapping task (UHFT) vs. control. The *t*-statistics map under ANOVA (uncorrected *P* < 0.001 and a cluster size of 10 voxels) were then determined and used for region of interest selection for the analysis of resting-state functional connectivity (RSFC).

For rs-fMRI, the BOLD signal of each voxel was first detrended to eliminate the linear trend and then a temporal band pass filter (0.01–0.08 Hz) was used to reduce low-frequency drift and high-frequency physiological noise. Finally, sources of spurious variance, including head motion parameters, white matter signals, and cerebrospinal signals, were removed by linear regression. A seed-based RSFC analysis was then calculated with voxel-wise Pearson’s correlation using the REST toolkit (24). The seed region was set as a spherical region (radius, 5.0 mm) centered at the peak activation point of the contralesional sensorimotor cortex, which was determined by the corresponding UHFT task-related functional map (Table S2 in Supplementary Material).

### Coupling between Motion-Related Activation and Resting-State Brain Activity

To describe the features of “coupling” between motion-related activation and resting-state brain activity, we investigated the relationship between these two modalities within the sensorimotor region. We combined voxels within the ipsilesional brain regions of task-evoked activation (*P* < 0.001 with FWE correction, one-sample *t*-test) and RSFC map (*P* < 0.001 with FWE correction, one-sample *t*-test) to create a group-specific mask (Figure S2 in Supplementary Material).

In accordance with previous studies performing across-modality analysis (25–27), we quantified coupling between the maps of motion-related activation and RSFC within the group-specific mask using across-voxel and across-subject correlation analyses, respectively. For cross-voxel correlation analysis, voxels from the motion-related activation map and RSFC map within the mask were correlated for each participant. This analysis measured the spatial similarity between these two maps and the correlation coefficient was regarded as the coupling strength. For the cross-subject correlation, analysis was carried out between patients at each time point and the healthy controls, respectively. Correlation was also carried out between motion-related activation maps and RSFC maps within the group-specific mask. These two maps were first standardized to *z*-scores and a correlation map was obtained for each participant group.

### Statistical Analysis

#### Dynamic Changes of Motion-Evoked Activation and rs-fMRI Activity on the Ipsilesional Side

The peak motion-related activation value of the AHFT and the peak RSFC strength, within the group-specific mask, were extracted for each patient. One-way repeated measures ANOVA and multiple comparisons (*post hoc* Tukey’s test) were used to investigate the difference between the UL-FMA scores, the peak motion-related activations, the peak RSFC strengths among the three stages using SPSS for Windows Version 22 (Armonk, NY, USA: IBM Corp.). Voxel-wise one-way repeated measures ANOVA and *post hoc* tests were also used to evaluate motor-related activation changes of the AHFT and RSFC changes among three stages using GLM Flex^2^. A two-sample *t* test was used to investigate the difference in peak motion-related activation, peak RSFC strength, and coupling strength within the group-specific mask between the health controls and stroke patients.

#### Dynamic Changes of Coupling Strengths between Task-Evoked and Resting-State Brain Activity on the Ipsilesional Side

One-way repeated measures ANOVA and multiple comparisons (*post hoc* Tukey’s test) were used to investigate the difference between the coupling strength among the three stages.

#### Correlations between Imaging Parameters and Behavioral Data

Pearson correlation analysis was used to investigate the relationships between functional imaging parameters (peak motion-related activation, peak RSFC strength, and coupling strength) and the UL-FMA score across the three stages.

## Results

### Clinical Data

There was a significant increase in UL-FMA scores during the longitudinal following (*F* = 68.55, *P* < 0.0001); furthermore, there were significant differences between all three stages (*P* < 0.0001).

### Dynamic Changes of Hand Motion-Related fMRI and rs-fMRI

**Figure 1:**
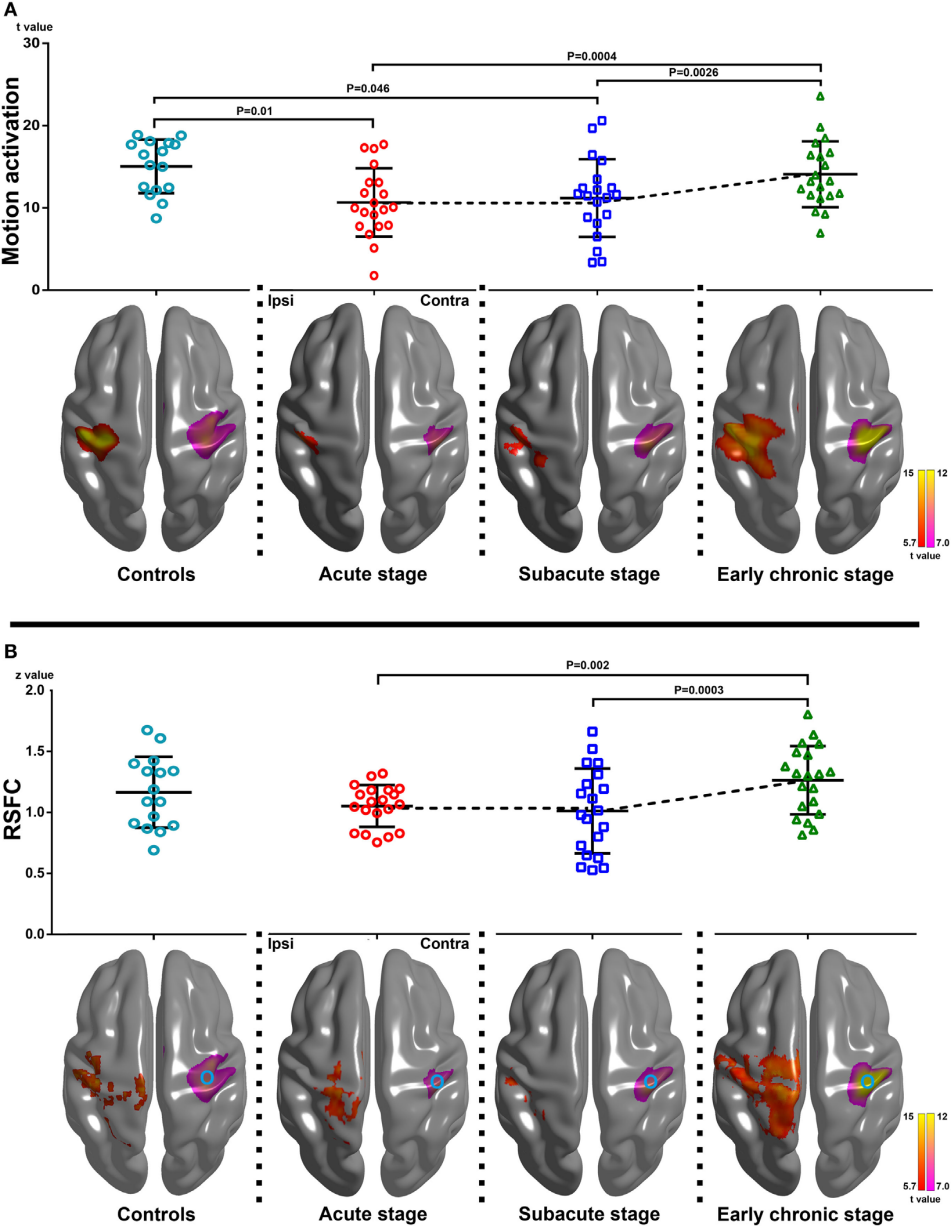
Changes of motion-related activation and resting-state functional connectivity (RSFC) in healthy controls and stroke patients across three stages of progression. **(A)** Upper row: peak motion-related activation changes within the group-specific mask across the three stages [*F* = 10.59, *P* = 0.0002, one-way repeated measures analysis of variance (ANOVA)]. Compared with the acute and subacute stages, peak motion-related activation increased significantly during the early chronic stage (*P* = 0.0004, *P* = 0.0026, respectively). Compared with healthy controls, stroke patients showed reduced motion-related activation and coupling strength during the acute and subacute stages (*P* = 0.01, *P* = 0.046, respectively; two-sample *t* test). Lower row: group motion-related activation maps of the affected hand finger tapping task (AHFT) and the unaffected hand finger tapping task (UHFT) in stroke patients during the three stages of progression and healthy controls (the right and left hand finger tipping task) (*P* < 0.05 with FEW correction). The group motion-related activation maps of the AHFT were located at the ipsilesional hemisphere, while the group motion-related activation maps of the UHFT were located at Contra. **(B)** Upper row: changes in the peak RSFC strength within the group-specific mask in healthy controls and stroke patients across the three stages of progression (*F* = 10.72, *P* = 0.0004, one-way repeated measures ANOVA). Compared with the acute and subacute stages, the peak RSFC strength increased significantly during the early chronic stage (*P* = 0.002, *P* = 0.0003, respectively). There were no significant differences in terms of peak RSFC strength between the healthy controls and stroke patients across the three stages of progression (two-sample *t* test). Lower row: group RSFC maps of the ipsilesional sensorimotor cortex across the three stages (*P* < 0.05 with FEW correction). The group RSFC maps of the ipsilesional sensorimotor cortex were located at Ipsi while the group motion-related activation maps of the UHFT were located at Contra. The circle in Contra represents the seed location of RSFC. Ipsi, the ipsilesional hemisphere; Contra, the contralesional hemisphere.

**Figure 2:**
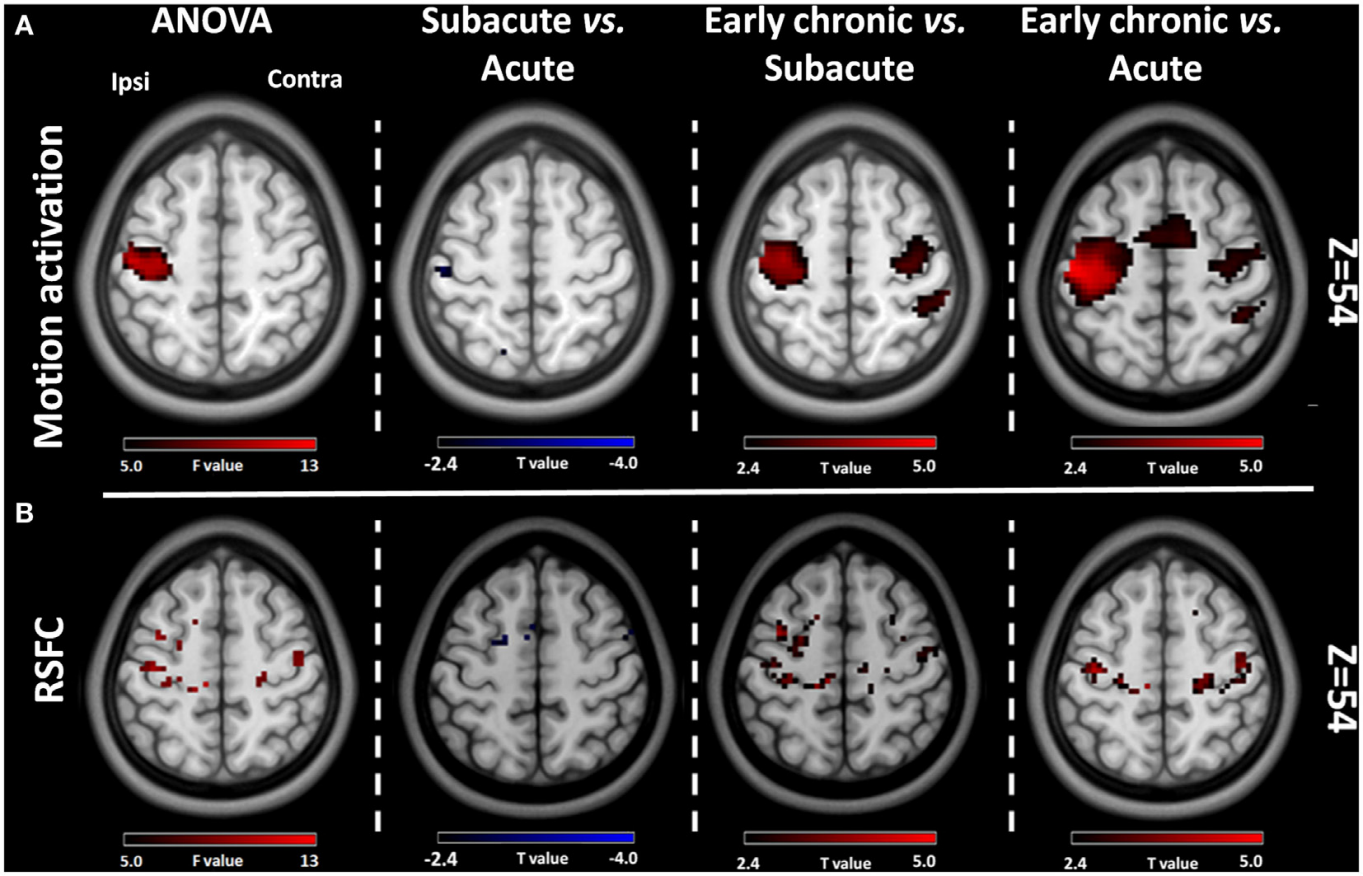
Comparative results of voxel-wise one-way repeated measures analysis of variance (ANOVA) (*P* < 0.001) and *post hoc* testing (*P* < 0.01) for motion-related activation and resting-state functional connectivity (RSFC) across the three stages of progression. **(A)** Motion-related activation results. Compared with the acute stage, motion-related activation was reduced in the ipsilesional sensorimotor cortex during the subacute stage. Compared with the acute and subacute stages, motion-related activation increased significantly during the early chronic stage. **(B)** RSFC results from the ipsilesional sensorimotor cortex. Compared with the acute stage, the RSFC of the ipsilesional sensorimotor cortex was reduced during the subacute stage. Compared with the acute and subacute stages, the FC of the ipsilesional sensorimotor cortex increased significantly during the early chronic stage. Ipsi, the ipsilesional hemisphere; Contra, the contralesional hemisphere.

The group motion-related activation maps of the AHFT and UHFT at each stage (*P* < 0.05 with FEW correction, one-sample *t* test) and the results of the peak motion-related activation of the AHFT among the three different stages are shown in Figure 1A. There was a significant difference in the peak motion-related activation of AHFT among the three stages (*F* = 10.59, *P* = 0.0002). Compared with the acute stage and subacute stage, the peak motion-related activation increased significantly during the early chronic stage (*P* = 0.0004, *P* = 0.0026, respectively). The motion-related activation changes of the AHFT, based on voxel-wise one-way repeated measures ANOVA (*P* < 0.001) and *post hoc* testing (*P* < 0.01), are demonstrated in Figure 2A and Table 2. Compared with the acute stage, the motion-related activation was significantly reduced in the ipsilesional sensorimotor cortex during the subacute stage. Compared with the acute and subacute stages, motion-related activation significantly increased in the ipsilesional sensorimotor cortex during the early chronic stage. We further compared peak motion-related activation of the AHFT between healthy controls and patients across the three stages. Compared with healthy controls, stroke patients showed significantly reduced motion-related activation during the acute and subacute stages (*P* = 0.01, *P* = 0.04, respectively).

**Table 2:** Comparing results of the motion-related activation and RSFC of the ipsilesional sensorimotor cortex among three stages.

	Motion-related activation						RSFC					
	Peak MNI coordinate (mm)			Peak	Peak	Cluster size	Peak MNI coordinate (mm)			Peak	Peak	Cluster size
					
	*X*	*Y*	*Z*	*F* value	*T* value		*X*	*Y*	*Z*	*F* value	*T* value	
ANOVA	−33	−21	51	14.72		273	−30	−24	63	10.03		20
Subacute vs. acute	−48	−24	60		−2.73	19	−27	0	60		−3.60	7
Early chronic vs. subacute	−30	−21	51		4.31	769	−30	−24	57		4.31	113
Early chronic vs. acute	−48	−18	57		5.32	1,338	−39	−18	54		3.79	72

*MNI, Montreal Neurological Institute; Cluster size, significant clusters within the group-specific mask; RSFC, resting-state functional connectivity; ANOVA, analysis of variance*.

The group RSFC maps of the ipsilesional sensorimotor cortex at each stage (*P* < 0.05 with FEW correction, one-sample *t* test), and the results of peak RSFC strength across the three stages, are shown in Figure 1B. There were significant differences in peak RSFC strength across the three stages (*F* = 10.72, *P* = 0.0004). Compared with the acute and subacute stages, peak RSFC increased significantly during the early chronic stage (*P* = 0.002, *P* = 0.0003, respectively). The changes in RSFC strength of the ipsilesional sensorimotor cortex, based on voxel-wise one-way repeated measures ANOVA (*P* < 0.001) and *post hoc* testing (*P* < 0.01), are demonstrated in Figure 2B and Table 2. Compared with the acute stage, the RSFC of the ipsilesional sensorimotor cortex significantly decreased during the subacute stage. Compared with the acute and subacute stages, the RSFC of the ipsilesional sensorimotor cortex increased significantly during the early chronic stage. Notably, peak RSFC strength did not show any significant difference between healthy controls and patients within each of the three stages.

### Dynamic Changes of Coupling Strength between Motion-Related Activation and Resting-State Brain Activity

**Figure 3:**
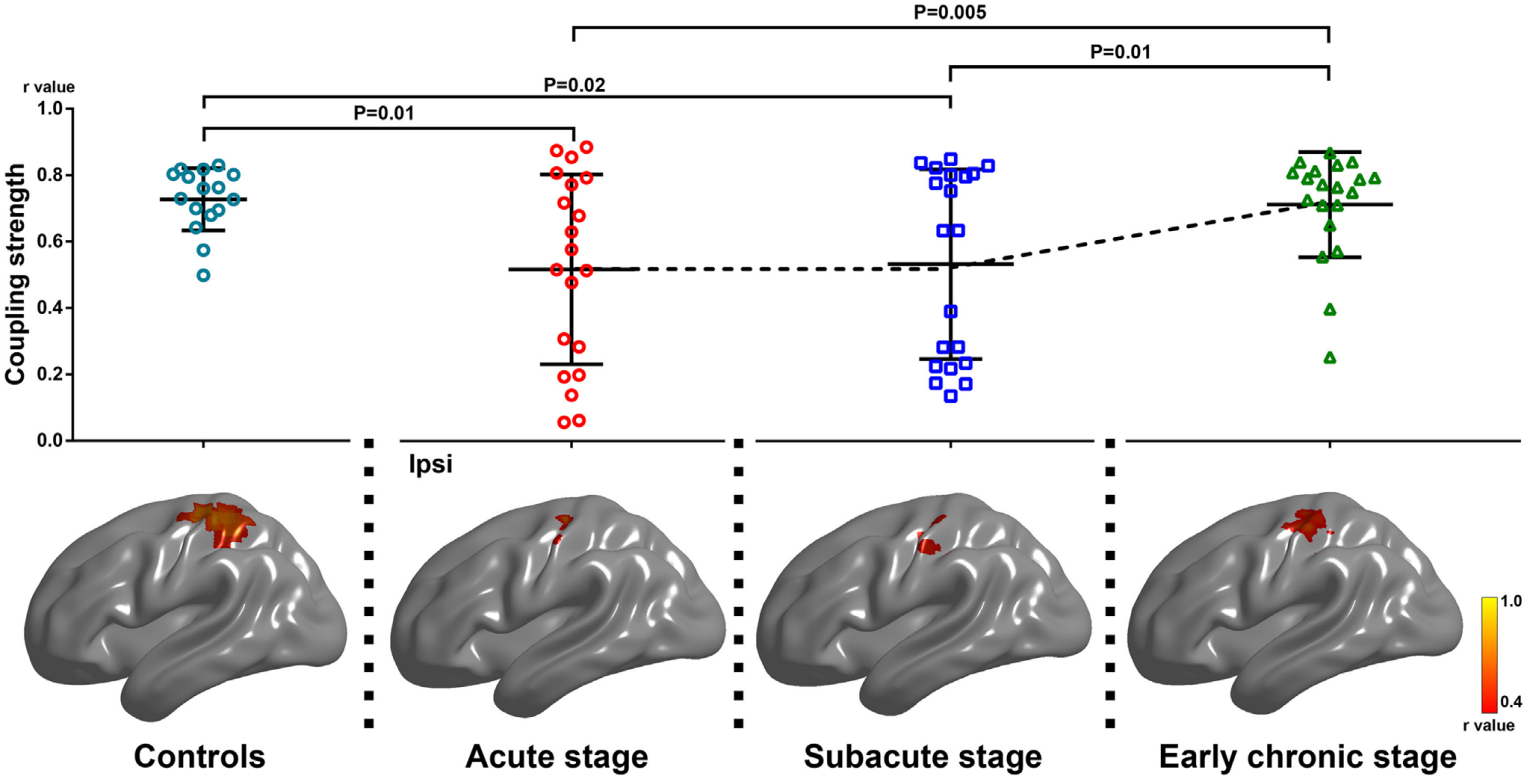
Alterations of coupling strength between motion-related activation and resting-state functional connectivity within the group-specific mask in healthy controls and stroke patients among three stages of progression. Upper row: changes in coupling strength across the three stages (*F* = 6.81, *P* = 0.003, one-way repeated measures analysis of variance). Compared with the acute and subacute stages, the degree of coupling increased significantly during the early chronic stage (*P* = 0.005, *P* = 0.01, respectively; *post hoc* test). Compared with healthy controls, stroke patients showed reduced motion-related activation and coupling strength during the acute and subacute stages (*P* = 0.01, *P* = 0.02, respectively; two-sample *t* test). Lower row: the results arising from our cross-subject correlation analysis within the group-specific mask in healthy controls and stroke patients across the three stages of progression. The level of significant voxel clearly increased during the early chronic stage. Ipsi, the ipsilesional hemisphere.

The coupling strength of the ipsilesional sensorimotor cortex at each time point was 0.52 ± 0.29, 0.54 ± 0.29, and 0.72 ± 0.16, respectively. The coupling strength increased significantly following motor function recovery (*F* = 6.81, *P* = 0.003). Compared with the acute and subacute stages, the coupling strength increased significantly during the early chronic stage (*P* = 0.005, *P* = 0.01, respectively). In addition, compared with the healthy controls, stroke patients showed reduced coupling during the acute and subacute stages (*P* = 0.01, *P* = 0.02, respectively, Figure 3).

The results of the cross-subject correlation analysis for the three stages are shown in Figure 3 (*P* < 0.05). Compared with the acute and subacute stages, the level of significant correlation between voxels of the ipsilesional sensorimotor mask clearly increased during the early chronic stage (Table 3).

**Table 3:** The results of across-subject correlation analysis at the three stages.

	Peak MNI coordinate (mm)			Peak *r* value	Cluster size
	*X*	*Y*	*Z*		
Acute stage	−27	−24	54	0.67	68
Subacute stage	−39	−24	60	0.73	143
Early chronic stage	−36	−27	54	0.76	222

*MNI, Montreal Neurological Institute; Cluster size, significant clusters within the group-specific mask*.

### Relationships between Functional Imaging Parameters and Behavioral Data

**Figure 4:**
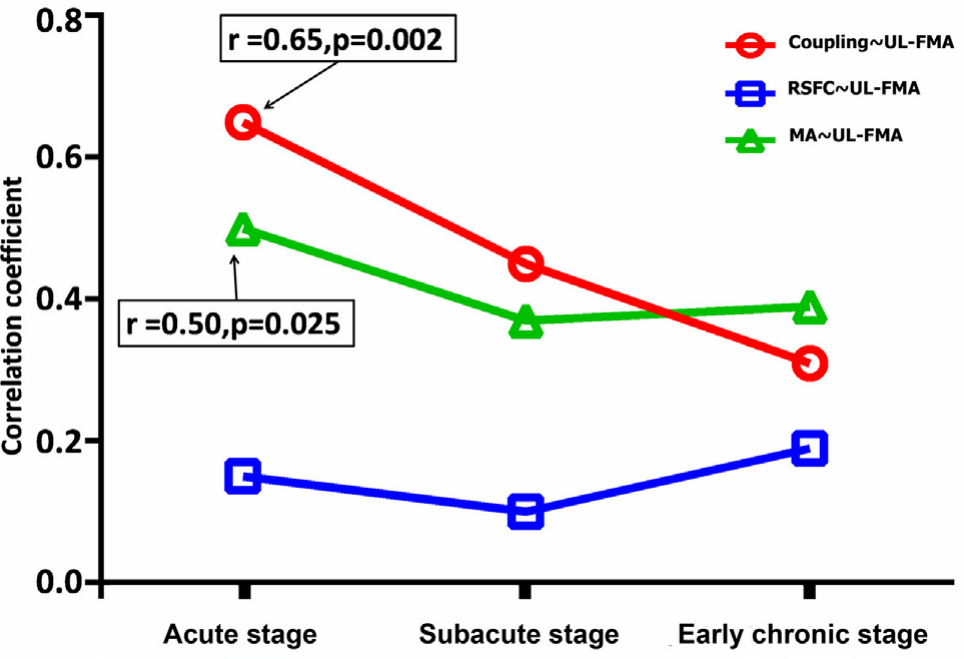
Alterations in the correlation coefficient between functional parameters [peak motion-related activation, peak resting-state functional connectivity (RSFC), and coupling strength] and Upper Limb Fugl-Meyer Assessment (UL-FMA) at the corresponding stage of progression. The peak motion-related activation and coupling strength during the acute stage was positively correlated to the UL-FMA scores at the corresponding stage (*r* = 0.50, 0.65; *P* = 0.025, *P* = 0.002, respectively). Red: correlation coefficient alteration between coupling strength and UL-FMA; green: correlation coefficient alteration between peak motion-related activation and UL-FMA; and blue: correlation coefficient alteration between peak RSFC and UL-FMA.

**Figure 5:**
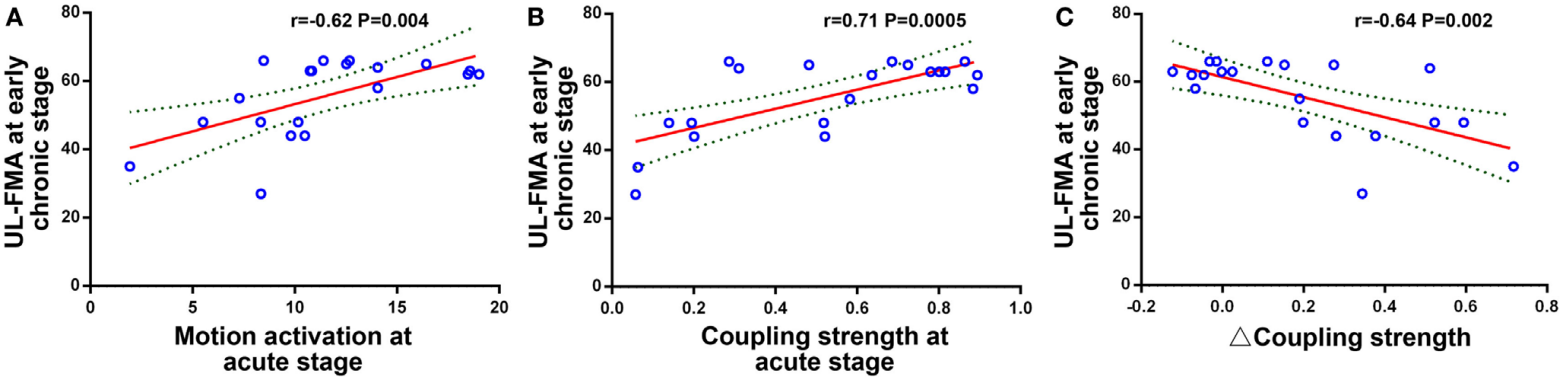
Relationship between functional imaging parameters and clinical outcome. **(A)** Peak motion-related activation during the acute stage was positively correlated to the Upper Limb Fugl-Meyer Assessment (UL-FMA) scores at the early chronic stage (*r* = 0.62, *P* = 0.004). **(B)** Coupling strength during the acute stage was positively correlated to the UL-FMA scores at the early chronic stage (*r* = 0.71, *P* = 0.0005). **(C)** The coupling strength change from the acute stage to the early chronic stage (Δ coupling strength) was negatively correlated to the UL-FMA scores during the early chronic stage (*r* = −0.64, *P* = 0.002).

Changes in the correlation coefficient between peak motion-related activation, peak RSFC strength, coupling strength, and the UL-FMA score during the three different stages are shown in Figure 4. The peak motion-related activation and coupling strength during the acute stage was positively correlated to UL-FMA scores within the corresponding stage (*r* = 0.50, 0.65; *P* = 0.025, *P* = 0.002, respectively). In addition, we found that motion-related activation and coupling strength during the acute stage was positively correlated to UL-FMA scores during the early chronic stage (*r* = 0.62, 0.71; *P* = 0.004, *P* = 0.0005, respectively; Figures 5A,B) and that the change in coupling strength from the acute stage to the early chronic stage (the coupling degree at early chronic stage minus that at the acute stage; Δcoupling strength) was negatively correlated to the UL-FMA scores during the early chronic stage (*r* = −0.64, *P* = 0.002, Figure 5C).

## Discussion

This study, for the first time, described the relationship between hand motion induced activation and RSFC in stroke patients by combining task-based fMRI and rs-fMRI. Our main novel findings were as follows: (1) compared with healthy controls, stroke patients showed reduced motion-related activation and coupling strength during the acute and subacute stages; (2) compared with the acute stage, motion-related activation and RSFC strength significantly increased in the ipsilesional sensorimotor cortex during the early chronic stage; and (3) coupling strength between motion-related activation and RSFC in the ipsilesional sensorimotor cortex was significantly increased following motor function recovery from the acute stage to the early chronic stage in stroke patients with motor impairment. Moreover, alterations in coupling strength were associated with motor recovery. Collectively, these findings indicated that the coupling relationship between task-based fMRI and rs-fMRI in stroke patients was influenced by different stages of pathological progression.

As two main functional imaging parameters, task-based fMRI and rs-fMRI can both provide useful information relating to cortical reorganization in stroke patients. In accordance with previous research (28–33), our results also demonstrated that the motion-related activation and RSFC strength of the ipsilesional sensorimotor cortex reduced during the subacute stage but significantly increased during the early chronic stage following the improvement of motor function. However, very little research has evaluated the specific relationship between motion-related activation and RSFC strength across different pathological stages in stroke patients by combining rs-fMRI and task-based fMRI.

In this study, a change in time-dependent coupling was identified between motion-related activation and RSFC in the ipsilesional sensorimotor cortex following the recovery of hand motor function. This finding further strengthens the viewpoint that functional restoration of the ipsilesional sensorimotor cortex is a key process for effective recovery of motor function (29). The crucial role of the ipsilesional sensorimotor cortex in motor recovery following stroke has been illustrated in several previous studies. For example, previous research found that all direct functional connections to muscle originated from the ipsilesional motor cortex after recovery from subcortical stroke (34). In task-based longitudinal fMRI studies, changes in brain activation showed an initial shift to the contralesional sensorimotor cortex but then returned to the ipsilesional sensorimotor cortex in stroke patients with better motor recovery (28–30, 35). rs-fMRI studies, based on human and animal research, also indicated that the RSFC between the ipsilesional primary motor cortex and the contralesional hemispheric cortex decreased as a result of motor deficit and that the RSFC of the ipsilesional primary motor area with other brain regions correlated with improvements in motor function (31, 33, 36–39).

Although the specific relationships between rs-fMRI and task-based fMRI still remain unclear, recent research has suggested that rs-fMRI and task-based fMRI signals might be governed by a common physiological mechanism (19). The temporal coherence of low-frequency rs-fMRI signals between brain regions reflects the possibility of a functional network of task-related activities within that region (6, 15), and that the amplitude of the rs-fMRI response is linearly related to task-fMRI responses (19). Moreover, one recent study demonstrated the close relationship between BOLD functional connectivity and neurovascular connections (40). Therefore, the coupling between motion-related activation and resting-state brain activity in the ipsilesional sensorimotor cortex might reflect sensorimotor network interaction between rs-fMRI and task-based fMRI. Dynamic alterations of coupling strength following stroke might, thus, reflect plastic alterations taking place due to impairment of the sensorimotor network.

Previous studies have indicated that the changes of functional imaging parameters in the first few days after stroke were sensitive enough to predict subsequent motor function outcome (41–43). Our results also revealed that the peak motion-related activation and coupling strength of the ipsilesional sensorimotor cortex during the acute stage were associated with motor function during the early chronic stages, while the increased coupling extent from the acute stage to the early chronic stage was negatively correlated to subsequent motor performance. In addition, we found that the coupling strength of the ipsilesional sensorimotor cortex was associated with task performance during the acute stage and that the correlation disappeared following motor recovery during the early chronic stage. Considering the degree of coupling degree almost returned to normal levels during the early chronic stage, we speculate that the extent of the change in coupling from the acute to the early chronic stage might reflect uncoupling degree between motion-related activation and resting-state brain activity, which might be related to the impairment level of the ipsilesional sensorimotor cortex. Although further studies are needed to verify the relationship between coupling strength and the level of impairment of the ipsilesional sensorimotor cortex, our current research indicates that coupling between motion-related activation and RSFC in the ipsilesional sensorimotor cortex can serve as a complementary measurement to assess motor impairment and clinical outcome.

There are some limitations to this study which need to be considered when interpreting our conclusions. First, although only patients with single stroke lesion within the territory of the middle cerebral artery were enrolled, the relative heterogeneity in lesion volume might have impacted upon the results of our study. Second, due to the differences in motor impairment, there may be disparity in terms of task performance for each patient. Although the task performance of each patient was visually monitored during the fMRI scan, inconsistencies in task performance may have influenced the results relating to motion-related activation. Third, although we adopted a longitudinal study design, a 3-month follow-up period, with three time points, is relatively short. A longer follow-up period, with a greater number of time points, will be helpful in fully elucidating the relationship between task-based fMRI and rs-fMRI in future studies.

In summary, in this study, we first illustrated the altered coupling between motion-related activation and RSFC in the ipsilesional sensorimotor cortex for stroke patients with hand motor impairment. Our findings demonstrated that coupling strength gradually increased following motor recovery after stroke. We also identified a clear linear relationship between the alterations in coupling patterns and motor recovery scores. These findings further extend our understanding of the role played by the ipsilesional sensorimotor cortex in the process of motor recovery following stroke. Generally, monitoring the coupling alteration between motion-related activation and RSFC in the ipsilesional sensorimotor cortex may be a complementary tool for evaluating and predicting motor recovery in stroke patients with motor impairment.

## Ethics Statement

This study was approved by the Internal Review Board of Jinling Hospital and written informed consent was obtained from each participant.

## Author Contributions

ZZ and GL conceived and designed the research. JD, JH, QX, FY, FZ, X-jD, and XL recruited the subjects, collected the data, performed the analysis, and generated the images. JH and ZZ wrote the paper. All authors read and approved the final draft.

## Conflict of Interest Statement

The authors declare that the research was conducted in the absence of any commercial or financial relationships that could be construed as a potential conflict of interest.

## Footnotes

^1^http://www.fil.ion.ucl.ac.uk/spm.

^2^http://nmr.mgh.harvard.edu/harvardagingbrain/People/AaronSchultz/GLM_Flex.html.

## References

[1] Tharin S Golby A. Functional brain mapping and its applications to neurosurgery. Neurosurgery (2007) 60:185–202. 10.1227/01.neu.0000255386.95464.52 17415154

[2] Hirsch J Ruge MI Kim KHS Correa DD Victor JD Relkin NR An integrated functional magnetic resonance imaging procedure for preoperative mapping of cortical areas associated with tactile, motor, language, and visual functions. Neurosurgery (2000) 47(3):711–22. 10.1097/00006123-200009000-00037 10981759

[3] Biswal BB Mennes M Zuo XN Gohel S Kelly C Smith SM Toward discovery science of human brain function. Proc Natl Acad Sci U S A (2010) 107(10):4734–9. 10.1073/pnas.0911855107 20176931 PMC2842060

[4] Guerra-Carrillo B Mackey AP Bunge SA. Resting-state fMRI: a window into human brain plasticity. Neuroscientist (2014) 20(5):522–33. 10.1177/1073858414524442 24561514

[5] Buckner RL Krienen FM Yeo BTT. Opportunities and limitations of intrinsic functional connectivity MRI. Nat Neurosci (2013) 16(7):832–7. 10.1038/nn.3423 23799476

[6] Murphy K Birn RM Bandettini PA. Resting-state fMRI confounds and cleanup. Neuroimage (2013) 80:349–59. 10.1016/j.neuroimage.2013.04.001 23571418 PMC3720818

[7] Fox MD Raichle ME Spontaneous fluctuations in brain activity observed with functional magnetic resonance imaging. Nat Rev Neurosci (2007) 8(9):700–11. 10.1038/nrn2201 17704812

[8] Kokkonen S-M Nikkinen J Remes J Kantola J Starck T Haapea M Preoperative localization of the sensorimotor area using independent component analysis of resting-state fMRI. Magn Reson Imaging (2009) 27(6):733–40. 10.1016/j.mri.2008.11.002 19110394

[9] Hou BL Bhatia S Carpenter JS. Quantitative comparisons on hand motor functional areas determined by resting state and task BOLD fMRI and anatomical MRI for pre-surgical planning of patients with brain tumors. Neuroimage Clin (2016) 11:378–87. 10.1016/j.nicl.2016.03.003 27069871 PMC4810013

[10] Hart MG Price SJ Suckling J Functional connectivity networks for preoperative brain mapping in neurosurgery. J Neurosurg (2017) 126(6):1941–50. 10.3171/2016.6.JNS1662 27564466

[11] Rosazza C Aquino D D’Incerti L Cordella R Andronache A Zaca D Preoperative mapping of the sensorimotor cortex: comparative assessment of task-based and resting-state FMRI. PLoS One (2014) 9(6):e98860. 10.1371/journal.pone.0098860 24914775 PMC4051640

[12] Zhang D Johnston JM Fox MD Leuthardt EC Grubb RL Chicoine MR Preoperative sensorimotor mapping in brain tumor patients using spontaneous fluctuations in neuronal activity imaged with functional magnetic resonance imaging: initial experience. Neurosurgery (2009) 65(6 Suppl):226–36. 10.1227/01.NEU.0000350868.95634.CA 19934999 PMC2796594

[13] Barkhof F Haller S Rombouts SA Resting-state functional MR imaging: a new window to the brain. Radiology (2014) 272(1):29–49. 10.1148/radiol.14132388 24956047

[14] Mannfolk P Nilsson M Hansson H Stahlberg F Fransson P Weibull A Can resting-state functional MRI serve as a complement to task-based mapping of sensorimotor function? A test-retest reliability study in healthy volunteers. J Magn Reson Imaging (2011) 34(3):511–7. 10.1002/jmri.22654 21761469

[15] Smith SM Fox PT Miller KL Glahn DC Fox PM Mackay CE Correspondence of the brain’s functional architecture during activation and rest. Proc Natl Acad Sci U S A (2009) 106(31):13040–5. 10.1073/pnas.0905267106 19620724 PMC2722273

[16] Fox MD Greicius M. Clinical applications of resting state functional connectivity. Front Syst Neurosci (2010) 4:19. 10.3389/fnsys.2010.00019 20592951 PMC2893721

[17] Tomasi D Wang R Wang GJ Volkow ND. Functional connectivity and brain activation: a synergistic approach. Cereb Cortex (2014) 24(10):2619–29. 10.1093/cercor/bht119 23645721 PMC4229895

[18] Mennes M Kelly C Zuo XN Di Martino A Biswal BB Castellanos FX Inter-individual differences in resting-state functional connectivity predict task-induced BOLD activity. Neuroimage (2010) 50(4):1690–701. 10.1016/j.neuroimage.2010.01.002 20079856 PMC2839004

[19] Kannurpatti SS Rypma B Biswal BB. Prediction of task-related BOLD fMRI with amplitude signatures of resting-state fMRI. Front Syst Neurosci (2012) 6:7. 10.3389/fnsys.2012.00007 22408609 PMC3294272

[20] Logothetis NK. The underpinnings of the BOLD functional magnetic resonance imaging signal. J Neurosci (2003) 23(10):3963–71. 12764080 10.1523/JNEUROSCI.23-10-03963.2003 PMC6741096

[21] Chao-Gan Y Yu-Feng Z DPARSF: a MATLAB toolbox for “pipeline” data analysis of resting-state fMRI. Front Syst Neurosci (2010) 4:13 10.3389/fnsys.2010.00013 20577591 PMC2889691

[22] Stebbins GT Nyenhuis DL Wang C Cox JL Freels S Bangen K Gray matter atrophy in patients with ischemic stroke with cognitive impairment. Stroke (2008) 39(3):785–93. 10.1161/STROKEAHA.107.507392 18258824

[23] Wei W Zhang Z Xu Q Yang F Sun K Lu G. More severe extratemporal damages in mesial temporal lobe epilepsy with hippocampal sclerosis than that with other lesions: a multimodality MRI study. Medicine (2016) 95(10):e3020. 10.1097/MD.0000000000003020 26962820 PMC4998901

[24] Song XW Dong ZY Long XY Li SF Zuo XN Zhu CZ REST: a toolkit for resting-state functional magnetic resonance imaging data processing. PLoS One (2011) 6(9):e25031 10.1371/journal.pone.0025031 21949842 PMC3176805

[25] Zhang Z Liao W Chen H Mantini D Ding JR Xu Q Altered functional-structural coupling of large-scale brain networks in idiopathic generalized epilepsy. Brain (2011) 134(Pt 10):2912–28. 10.1093/brain/awr223 21975588

[26] Zhang Z Xu Q Liao W Wang Z Li Q Yang F Pathological uncoupling between amplitude and connectivity of brain fluctuations in epilepsy. Hum Brain Mapp (2015) 36(7):2756–66. 10.1002/hbm.22805 25879781 PMC6869589

[27] Liang X Zou Q He Y Yang Y Coupling of functional connectivity and regional cerebral blood flow reveals a physiological basis for network hubs of the human brain. Proc Natl Acad Sci U S A (2013) 110(5):1929–34. 10.1073/pnas.1214900110 23319644 PMC3562840

[28] Rehme AK Fink GR von Cramon DY Grefkes C. The role of the contralesional motor cortex for motor recovery in the early days after stroke assessed with longitudinal FMRI. Cereb Cortex (2011) 21(4):756–68. 10.1093/cercor/bhq140 20801897

[29] Rehme AK Eickhoff SB Rottschy C Fink GR Grefkes C. Activation likelihood estimation meta-analysis of motor-related neural activity after stroke. Neuroimage (2012) 59(3):2771–82. 10.1016/j.neuroimage.2011.10.023 22023742

[30] Ward NS Brown MM Thompson AJ Frackowiak RS. Neural correlates of motor recovery after stroke: a longitudinal fMRI study. Brain (2003) 126(Pt 11):2476–96. 10.1093/brain/awg245 12937084 PMC3717457

[31] Carter AR Astafiev SV Lang CE Connor LT Rengachary J Strube MJ Resting interhemispheric functional magnetic resonance imaging connectivity predicts performance after stroke. Ann Neurol (2010) 67(3):365–75. 10.1002/ana.21905 20373348 PMC2927671

[32] Carter AR Shulman GL Corbetta M. Why use a connectivity-based approach to study stroke and recovery of function? Neuroimage (2012) 62(4):2271–80. 10.1016/j.neuroimage.2012.02.070 22414990 PMC3733251

[33] Park CH Chang WH Ohn SH Kim ST Bang OY Pascual-Leone A Longitudinal changes of resting-state functional connectivity during motor recovery after stroke. Stroke (2011) 42(5):1357–62. 10.1161/STROKEAHA.110.596155 21441147 PMC3589816

[34] Mima T Toma K Koshy B Hallett M. Coherence between cortical and muscular activities after subcortical stroke. Stroke (2001) 32(11):2597–601. 10.1161/hs1101.098764 11692023

[35] Grefkes C Ward NS. Cortical reorganization after stroke: how much and how functional? Neuroscientist (2014) 20(1):56–70. 10.1177/1073858413491147 23774218

[36] Carter AR Patel KR Astafiev SV Snyder AZ Rengachary J Strube MJ Upstream dysfunction of somatomotor functional connectivity after corticospinal damage in stroke. Neurorehabil Neural Repair (2011) 26(1):7–19. 10.1177/1545968311411054 21803932 PMC3822763

[37] Rehme AK Eickhoff SB Wang LE Fink GR Grefkes C. Dynamic causal modeling of cortical activity from the acute to the chronic stage after stroke. Neuroimage (2011) 55(3):1147–58. 10.1016/j.neuroimage.2011.01.014 21238594 PMC8053821

[38] Wang L Yu C Chen H Qin W He Y Fan F Dynamic functional reorganization of the motor execution network after stroke. Brain (2010) 133(Pt 4):1224–38. 10.1093/brain/awq043 20354002

[39] van Meer MP van der Marel K Wang K Otte WM El Bouazati S Roeling TA Recovery of sensorimotor function after experimental stroke correlates with restoration of resting-state interhemispheric functional connectivity. J Neurosci (2010) 30(11):3964–72. 10.1523/JNEUROSCI.5709-09.2010 20237267 PMC6632290

[40] Tak S Polimeni JR Wang DJ Yan L Chen JJ. Associations of resting-state fMRI functional connectivity with flow-BOLD coupling and regional vasculature. Brain Connect (2015) 5(3):137–46. 10.1089/brain.2014.0299 25384681 PMC4394176

[41] Rehme AK Volz LJ Feis DL Eickhoff SB Fink GR Grefkes C. Individual prediction of chronic motor outcome in the acute post-stroke stage: behavioral parameters versus functional imaging. Hum Brain Mapp (2015) 36(11):4553–65. 10.1002/hbm.22936 26381168 PMC4619153

[42] Price CJ Hope TM Seghier ML Ten problems and solutions when predicting individual outcome from lesion site after stroke. Neuroimage (2017) 145(Pt B):200–8. 10.1016/j.neuroimage.2016.08.006 27502048 PMC5154378

[43] Rehme AK Volz LJ Feis DL Bomilcar-Focke I Liebig T Eickhoff SB Identifying neuroimaging markers of motor disability in acute stroke by machine learning techniques. Cereb Cortex (2015) 25(9):3046–56. 10.1093/cercor/bhu100 24836690

