# Supplementary material for: Altered Coupling between Motion-Related Activation and Resting-State Brain Activity in the Ipsilesional Sensorimotor Cortex after Cerebral Stroke
Source: Front Neurol. 2017 Jul 19;8:339. doi: 10.3389/fneur.2017.00339 (PMC5515815; doi:10.3389/fneur.2017.00339)
Supplement: Supplementary file 1 [file Data_Sheet_1.PDF]

Supplementary Material

Dynamically changed prediction of resting-state brain activity for motion-related activation in cerebral stroke

Jianping Hu[1,2,3†], Juan Du[4†] , Qiang Xu[3], Fang Yang[4], Fanyong Zeng[3], Xi-jian Dai[3],Xiaoxue Liu[3], Zhiqiang Zhang [3]*, Guangming Lu [1,3]*

*** Correspondence:** Guangming Lu cjr.luguangming@vip.163.com， Zhiqiang Zhang zhangzq2001@126.com

† These authors have contributed equally to this work

1 Supplementary Figures and Tables

1.1 Supplementary Figure

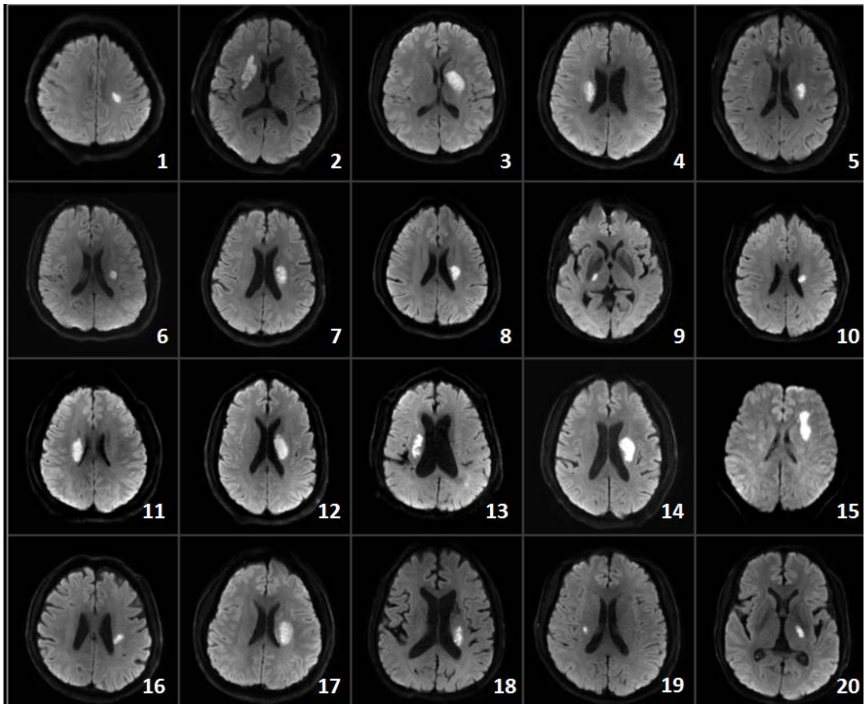

Supplementary Figure 1. The lesion locations of stroke patients. The lesions are shown on axial slices of the diffusion-weighted images.

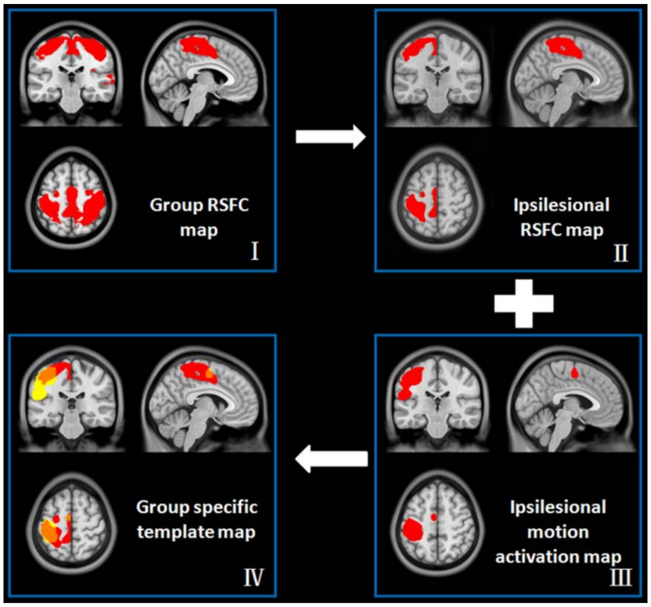

Supplementary Figure 2. The group-specific mask establishment of the ipsilesional sensorimotor cortex.(I) The general group resting state functional connectivity (RSFC) map of the ipsilesional sensorimotor cortex based on a seed derived from the unaffected hand movement task fMRI. (II) The ipsilesional side of the general RSFC mapping was extracted as the ipsilesional sensorimotor cortex by masking the contralesional sensorimotor cortex. (III) The general group activity map of the ipsilesional sensorimotor cortex based on the uffected hand finger tapping task (UHFT) fMRI.(IV) two ipsilesional sensorimotor cortex based on the activity map (III)and FC map(II) were merged as the group-specific mask of the ipsilesional sensorimotor cortex(IV).

1.2 Supplementary Tables

Supplemental Table1. Demograpic and clinical data of stroke patinents.

**Patient ID**	**Gender**	**Age (years)**	**Lesion Side**	**Lesion Location**	**Lesion Volume (ml)**	**Days after stroke**			**UL-FMA score**		
						**TP1**	**TP2**	**TP3**	**TP1**	**TP2**	**TP3**
**1**	M	44	L	CR	0.89	2	7	97	53	61	66
**2**	M	50	R	BG	6.35	2	8	113	33	39	63

**3**	F	45	L	BG	6.08	3	8	94	59	62	66
**4**	M	50	R	BG	3.54	6	12	103	56	61	64
**5**	M	49	L	BG	3.99	7	13	108	46	57	62
**6**	M	43	L	CR	1.90	3	9	93	48	58	65
**7**	M	61	L	CR	5.80	6	12	112	40	43	55
**8**	M	39	L	BG	2.41	3	10	94	38	49	63
**9**	F	57	R	IC	0.75	1	8	94	35	47	63
**10**	M	65	L	CR	0.87	3	9	95	40	53	62
**11**	M	35	R	CR	4.56	3	8	116	6	7	35
**12**	M	57	L	CR	4.93	3	9	92	31	38	44
**13**	F	64	R	BG	3.23	4	9	100	26	33	48
**14**	M	52	L	CR	4.57	4	9	105	12	12	48
**15**	M	30	L	BG	5.65	3	14	97	40	58	66
**16**	M	56	L	CR	1.54	3	14	91	48	51	58
**17**	M	32	L	BG	6.44	6	11	87	14	28	44
**18**	M	71	L	BG	1.96	4	9	92	36	39	48
**19**	M	61	R	CR	0.61	3	8	90	51	61	65
**20**	M	58	L	IC	1.21	6	12	93	10	15	27
**Mean (SD)**		50.95(11.40)			3.36(2.08)	3.75(1.62)	9.95(2.16)	98.3 (8.39)	36.1(15.65)	43.60(17.18)	55.60(11.49)

Abbreviations: CR, corona radiate; BG, basal ganglia; IC, internal capsule; SD, standard deviation; TP, time point;TP1,acute stage,TP2,subacute stage; TP3,early chronic stage ; UL-FMA, upper limb Fugl-Meyer Assessment.

Supplemental Table2. The peak Montreal Neurological Institute (MNI) coordinates and intensity of the intact hand motion-related activation maps that were used to be the seed points.

**Patient NO.**	**Acute stage**	**Subacute stage**	**Early chronic stage**
	Peak MNI coordinates(mm)	Peak MNI coordinates(mm)	Peak MNI coordinates(mm)
	x y z	x y z	x y z
**1**	33 -27 66	36 -24 57	36 -21 54
**2**	36 -15 72	45 -27 51	36 -21 66
**3**	36 -21 51	30 -21 60	33 -24 66
**4**	36 -27 66	33 -45 69	36 -27 69
**5**	39 -18 63	39 -18 51	42 -30 66
**6**	39 -42 66	33 -33 72	36 -18 48
**7**	39 -18 69	39 -12 69	42 -15 66
**8**	45 -27 63	48 -18 54	45 -39 60
**9**	33 -21 69	30 -24 72	33 -24 72
**10**	48 -30 63	42 -12 48	45 -3 57
**11**	45 -24 63	51 -30 60	39 -24 63
**12**	36 -21 69	36 -18 66	36 -15 63
**13**	36 -24 69	39 -30 66	57 -18 51

**14**	24 -30 75	54 -15 57	36 -18 63
**15**	48 -9 57	36 -39 66	39 -33 66
**16**	45 -27 60	39 -18 66	39 -24 60
**17**	33 -42 66	63 -21 45	63 -6 42
**18**	39 -24 63	33 -21 48	45 -15 57
**19**	39 -18 69	39 -30 72	39 -18 51
**20**	36 -24 63	48 -24 60	42 -30 63

MNI: Montreal Neurological Institute
